# Noncoding RNAs in atherosclerosis: regulation and therapeutic potential

**DOI:** 10.1007/s11010-023-04794-0

**Published:** 2023-07-07

**Authors:** Luyao Qi, Jixiang Xing, Yuesong Yuan, Ming Lei

**Affiliations:** 1 Critical Care Medicine, Seventh People’s Hospital of Shanghai University of Traditional Chinese Medicine, 200137 Shanghai, China; 2 First College of Clinical Medicine, Shandong University of Traditional Chinese Medicine, 250014 Jinan, Shandong China; 3 Peripheral Vascular Department, The Second Affiliated Hospital of Tianjin University of Traditional Chinese Medicine, 300150 Tianjin, China

**Keywords:** Atherosclerosis, Noncoding RNAs, Endothelial, Smooth muscle cells, Therapy

## Abstract

Atherosclerosis, a chronic disease of arteries, results in high mortality worldwide as the leading cause of cardiovascular disease. The development of clinically relevant atherosclerosis involves the dysfunction of endothelial cells and vascular smooth muscle cells. A large amount of evidence indicates that noncoding RNAs, such as microRNAs (miRNAs), long noncoding RNAs (lncRNAs), and circular RNAs (circRNAs), are involved in various physiological and pathological processes. Recently, noncoding RNAs were identified as key regulators in the development of atherosclerosis, including the dysfunction of endothelial cells, and vascular smooth muscle cells and it is pertinent to understand the potential function of noncoding RNAs in atherosclerosis development. In this review, the latest available research relates to the regulatory role of noncoding RNAs in the progression of atherosclerosis and the therapeutic potential for atherosclerosis is summarized. This review aims to provide a comprehensive overview of the regulatory and interventional roles of ncRNAs in atherosclerosis and to inspire new insights for the prevention and treatment of this disease.

## Introduction

Atherosclerosis (AS) is a chronic inflammatory disease of the artery wall that is involved in a series of cardiovascular diseases (CVDs), including stroke, ischemic heart disease and peripheral vascular disease [1]. Investigating the molecular mechanisms that promote atherosclerosis formation and development are indispensable for finding new therapeutic strategies. Atherosclerosis initially starts from the dysfunction of endothelial and vascular smooth muscle cells, and then lipids accumulate in the artery wall, followed by persistent inflammation, finally causing plaque rupture and thrombosis [2]. Endothelial cells (ECs) and vascular smooth muscle cells (VSMCs) are crucial cells in the occurrence and progression of atherosclerosis, and understanding the regulatory mechanisms of these dysfunctional cells during the process of atherosclerosis will be significant for the treatment and prevention of this disease in the future.

ECs undergo migration in response to various stimuli, such as inflammatory mediators and hemodynamic forces, leading to the formation of neointima and plaque formation [3]. Altered endothelial adhesion properties, such as the upregulation of adhesion molecules like intercellular adhesion molecule-1 (ICAM-1) and vascular cell adhesion molecule-1 (VCAM-1), facilitate the recruitment and adhesion of leukocytes to the endothelial surface [4]. These interactions between endothelial cells and leukocytes contribute to the initiation and progression of AS. Endothelial lipid metabolism is also a key factor in AS pathogenesis. Dysregulated lipid homeostasis, including impaired uptake and processing of low-density lipoprotein (LDL), leads to the accumulation of lipid deposits within the arterial wall, promoting the formation of atherosclerotic plaques [5]. VSMCs play a crucial role in maintaining the structural integrity of blood vessels. However, in response to stimuli and growth factors, VSMCs can undergo phenotypic modulation, leading to excessive proliferation and migration, contributing to neointima formation and plaque development [6].

A large amount of evidence indicates that epigenetic modification plays a critical role in plaque progression and vulnerability, which highlights the potential role of therapeutics in atherosclerosis intervention [2]. Noncoding RNAs, including miRNAs, lncRNAs, and circRNAs, are emerging as crucial epigenetic regulators of pathophysiological processes during the development of atherosclerosis, such as cellular activation, adhesion, proliferation, and inflammatory factor production in ECs and VSMCs. Here, we summarize the latest findings for the molecular mechanisms of noncoding RNAs in regulating ECs and VSMCs during atherosclerosis and discuss their potential clinical applicability for atherosclerosis.

## Biology of noncoding RNAs

With the development of sequencing technology, an increasing number of meaningful noncoding RNAs have been found and investigated in a variety of diseases. MiRNAs, lncRNAs, and circRNAs are the most studied noncoding RNAs, show significant roles in regulating gene expression and have potential clinical applicability in disease intervention.

MiRNAs are characterized as a class of ∼ 22-nt-long nonprotein-coding RNAs derived from longer transcripts. In 1993, Lin-4 was the first miRNA to be identified from *Caenorhabditis elegans* as a regulator of gene expression controlling developmental timing [7]. Generally, miRNAs are initially derived from their own noncoding gene or from the introns of protein-coding genes in the nucleus as long primary transcripts (pri-miRNAs) and are further cleaved, exported, and processed in the cytoplasm [8]. Applying the method of biological and bioinformatics, many thousands of miRNAs have been discovered, and newly found miRNAs have been compiled officially in miRBase (http://www.mirbase.org/) [9], which consists of published miRNA sequences and annotations as well as novel miRNAs before publication. They possess the capacity to negatively regulate gene expression at the posttranscriptional level in a sequence-specific manner, mainly through base pairing to the 3′-untranslated region (3′ UTR) of target mRNA transcripts [10].

LncRNAs are characterized as > 200 nt in length and without an apparent protein-coding capacity, and they are derived from approximately 98–99% of the noncoding regions as well as exonic, intronic, and intergenic regions of the genome. Currently, there are eight categories of lncRNAs, including enhancer, intronic, promoter, intergenic, bidirectional, small nucleolar RNA-ended, natural antisense/sense, and nonpoly(A) lncRNAs [11–17]. A series of studies have indicated that lncRNAs can manipulate cellular functions, such as cell proliferation, apoptosis, migration, differentiation, and metabolism, by regulating the expression of target genes through diverse mechanisms. For example, lncRNAs can target DNA, RNAs, and/or proteins to regulate transcription, epigenetic modifications, translation, protein/RNA stability, and posttranslational modifications [18–21]. LncRNAs are mainly transcribed via RNA polymerase II and are polyadenylated [22], and the majority of lncRNAs are stabilized via polyadenylation or secondary structures [23]. LncRNAs can obstruct miRNAs and proteins to modulate their activity and levels, disturb posttranslational modification processes, or participate in mRNA translation and stability [24–26]. In addition, lncRNAs may play different functions by interacting with different targets according to the subcellular microenvironment [27].

CircRNAs exist in closed loop RNA structures that are biologically active and derived from mRNAs via spliceosome mediated back splicing: the 3′ splicing site is covalently linked to the 5′ splicing site [28]. It was first reported in RNA viruses as early as 1976 [29] and then verified as an endogenous RNA splicing product in eukaryotes in 1979 [30]. Subsequently, a series of circRNAs was documented, and dysregulated expression of circRNAs was found in various physiological conditions. CircRNAs consist of four categories: circular RNAs from introns, intergenic circRNAs, exonic circRNAs, and exon–intron circRNAs [31]. CircRNAs are generally treated as noncoding RNAs, but some circRNAs have the capacity to encode proteins [32]. A series of regulatory mechanisms of circRNA in pathophysiological processes were reported, such as those serving as scaffolds to promote the assembly of protein complexes, regulating parental gene expression, regulating the stability of mRNAs, modulating alternative splicing, interacting with RNA binding proteins, and competing with endogenous RNAs or miRNA sponges [33].

## Noncoding RNA regulation of endothelial cells

### Role of miRNAs in endothelial cells

AS, the pathological factor in cardiovascular disease, is treated as a lipid-driven inflammatory disorder of the arteries. Large amounts of evidence have indicated that miRNAs are involved in endothelial cell dysfunction in AS. Previously, Li reported that oxLDL has could stimulate miRNA-146a promoter activity in macrophages through the AP-1 and NF-κB pathways, which leads to the suppression of macrophage maturation [34]. Recently, Xiao et al. reported that miR-146a is upregulated both in endothelial cells and extracellular vesicles derived from mesenchymal stem cells. Furthermore, they found that miR-146a plays a critical role in inhibiting endothelial senescence by regulating the phosphorylation of Src and the expression of caveolin-1 and VE-cadherin [35]. In addition, miR-146a is dysregulated in several cancers as an oncogene involved in the development of cancer by regulating apoptosis and the proliferation of cancer cells [36]. These data suggest that miR-146a plays an important role in regulating the progression of a variety of diseases, and the regulatory mechanisms and linkages among different diseases require further elucidation.

It has been reported that miR-216a was upregulated in patients with coronary artery disease, and further investigation indicated that miR-216a could lead to endothelial cell senescence and inflammation by activating the Smad3/IκBα pathway [37]. Overexpression of miR-216a in human umbilical vein endothelial cells (HUVECs) can cause senescence and promote inflammation by suppressing Smad3 protein expression and improving IκBα degradation. In addition, miR-216a also promotes expression by activating the NF-κB pathway, which promotes monocyte adhesion to endothelial cells [38]. Ginsenoside Rb2 (Rb2), a traditional Chinese medicine extracted from the plant Panax ginseng, can specifically bind to miR-216a and attenuate senescence and inflammation induced by miR-216a, indicating that Rb2 could serve as a potential therapeutic drug for AS by targeting miR-216a [38].

Inflammation is a main contributor to the development of atherosclerosis. In recent years, a series of studies have shown that miRNAs play a crucial role in regulating endothelial inflammation and may serve as potential therapeutic targets for atherosclerosis intervention. Recently, Hou et al. found that miR-146a-5p overexpression attenuates the inflammatory response in HUVECs induced by lipopolysaccharides (LPS) by suppressing tumor necrosis factor receptor associated 6 (TRAF6) and interleukin-1-receptor-associated kinase 1 (IRAK1), which indicates that miRNA-146a-5p may serve as a potential therapeutic target for atherosclerosis [39].

It was reported that overexpression of miR-520b in ECs suppresses inflammatory gene expression, including intercellular adhesion molecule 1 (ICAM1), vascular cell adhesion molecule 1 (VCAM1), and selectin E (SELE), by targeting RELA [40]. Furthermore, miR-520b inhibits the adhesion of monocytes and monocyte trans-endothelial migration stimulated with LPS, which indicates the crucial role of miR-520b in the occurrence of AS and the therapeutic potential of atherosclerosis. However, a series of miRNAs could aggravate the inflammatory response in ECs and promote AS development.

Previously, Li et al. reported that miR-19b could promote atherosclerosis progression by augmenting perivascular adipose tissue-specific inflammation by repressing suppressor of cytokine signaling 3 (SOCS3) expression [41]. Recently, miR-19b was shown to be upregulated in oxLDL-treated HUVECs and arteries in an AS mouse model [42]. Mechanistically, miR-19b could target PPARγ and downregulate its expression, promoting inflammatory cytokine production, such as TNF-α and IL-1β, in the AS model, which indicates that miR-19b inhibition may serve as a potent therapeutic consideration for AS intervention.

Extracellular vesicles (EVs) have been reported to play a crucial role in endothelial inflammation and atherogenesis. Recently, Jing et al. found that hepatocyte-derived EVs promote the inflammatory response in ENs in the context of non-alcoholic fatty liver disease (NAFLD) [43]. Furthermore, they disclosed that miR-1 in EVs could improve endothelial inflammation by inhibiting the expression of kruppel like factor 4 (KLF4) and finally facilitating atherogenesis development.

The abnormal proliferation of ECs induced during vascular injury is a pathological basis of atherosclerosis. It has been reported that miR-15a-5p plays an important role in sepsis via manipulating the inflammatory related signaling pathway [44]. Recently, miR-15a-5p was found to inhibit vascular endothelial cell proliferation by suppressing CX3CL1 transcription and affecting atherosclerosis progression [45].

Gomez et al. reported that neutrophil-derived microvesicles can promote atherosclerotic plaque formation and the inflammatory response by delivering miR-155, which suggests a critical role of miR-155 in AS progression [46]. Furthermore, the expression of miR-155 is upregulated in polycyclic aromatic hydrocarbon (PAH)-stimulated HUVECs and has been implicated in increased permeability and decreased proliferation [47]. Mechanistically, miR-155 directly targets serpin family D member 1 (SERPIND1), downregulates its expression and causes endothelial injury. The above research indicates that miR-155 is a multifunctional regulatory factor in the process of AS and may be treated as a potential drug target for AS treatment in the future.

Mesenchymal stem cell (MSC)-derived miRNAs play an important role in regulating cell functions, especially ECs. Chen et al. reported that miR-512-3p, enriched in exosomes derived from MSCs, can protect ECs from oxLDL-mediated damage by promoting their proliferation, inhibiting apoptosis, suppressing the expression of inflammatory cytokines and oxidative factors, and increasing the contents of superoxide dismutase (SOD) and glutathione peroxidase (GSH-PX). Mechanistic studies have shown that miR-512-3p targets Keleh-like ECH-associated protein 1 (Keap1) directly to protect ECs against oxLDL stimulation [48]. However, Ge et al. reported that miR-512-3p expression is upregulated in oxLDL-treated HUVECs and that silencing of miR-512-3p can attenuate apoptosis and ER stress and promote the proliferation and viability of HUVECs stimulated with oxLDL [49]. Furthermore, they found that miR-512-3p can target X-box binding protein 1 (XBP-1) to regulate the ratio of XBP-1S/XBP-1U to participate in atherosclerotic lesions. It is possible that the source of miRNA determines the physiological functions for the regulation of ECs cells during the AS process.

Angiogenesis is implicated in a variety of biological processes, including AS and cancer. Dickkopf1 (DKK1) plays many roles in both tumors and AS and has emerged as a potential biomarker of cancer progression and prognosis. Di et al. reported that DKK1 plays a critical role in AS progression by promoting plaque formation and vulnerability by causing EC apoptosis [50]. Baetta et al. also documented that DKK1 is a key driver of the initiation and progression of AS [51]. Recently, DKK1 was reported to be involved in angiogenesis both in the plaques of ApoE^−/−^ mice and oxLDL-treated HUVECs [52]. Mechanistically, the authors found that miR33a-5p was significantly downregulated in oxLDL-stimulated HUVECs and that miR33a-5p could directly bind to the 3′-UTR of Ets-1 to manipulate the expression of Ets-1 and DKK1 and inhibit EC migration and angiogenesis.

Low shear stress and pyroptosis play key roles in the initiation and progression of AS. MiR-181b-5p is downregulated under shear stress in HUVECs, accompanying the upregulation of the NLR family pyrin domain containing 3 (NLRP3) inflammasome, and re-expression of miR-181b-5p attenuates NLRP3 inflammasome-induced pyroptosis [53]. Mechanistically, miR-181b-5p can target the 3′-UTR of the signal transducer and activator of transcription 3 (STAT-3) gene, inhibit NLRP3 inflammasome expression, and alleviate shear stress-induced pyroptosis, which indicates the crucial role of miR-181b-5p in the treatment of AS. In addition, miR-18b is downregulated in AS plaques compared with control arterial tissues, and miR-181b participates in the formation of AS plaques and injury of vascular ECs by targeting notch receptor 1 (Notch1) and downregulating its expression [54]. However, Gregoli et al. found that miR-181b is upregulated in symptomatic human atherosclerotic plaques and abdominal aortic aneurysms [55]. Furthermore, they disclosed that miR-181b can maintain atherosclerotic plaque and aneurysm stability by regulating TIMP metallopeptidase inhibitor 3 (TIMP-3) and elastin expression, which indicates that miR-181b is a key regulator in suppressing atherosclerosis and aneurysm progression and protecting plaque rupture, and it could be a potential therapeutic option for cardiovascular disease intervention in the future. Previously, butyrate has been reported to play beneficial roles in cardiovascular diseases by preventing endothelial injury in atherosclerosis, but the underlying mechanisms remain unknown. Recently, miR-181b expression was reported to be upregulated in atherosclerotic aortas and IL-1β-treated ECs after treatment with butyrate [56]. Importantly, the increased miR-181b induced by butyrate was shown to inhibit NADPH oxidase 2 (NOX2) expression and reactive oxygen species (ROS) generation in ECs.

### Role of lncRNAs in endothelial cells

EC dysfunction is one of the main reasons for the occurrence of chronic disorders, including atherosclerosis, and is highly related to the abnormal expression of lncRNAs. LncRNA PVT1, located on human chromosome 8q24 adjacent to the c-myc genes, is considered a potential oncogene. Indeed, a series of studies have reported that lncRNA PVT1 expression is increased in many malignancies and is involved in promoting tumor cell proliferation, migration, and metastasis [56]. Recently, lncRNA PVT1 was found to be upregulated in the serum of AS patients and in oxLDL-stimulated HUVECs, and repression of lncRNA PVT1 in oxLDL-treated HUVECs promotes proliferation and inhibits apoptosis and production of inflammatory cytokines by downregulating the expression of miR-30c-5p [57].

It has been reported that lncRNA NEAT1 is involved in regulating gastric progression by manipulating the miR‐335‐5p/ROCK1 pathway [58]. In addition, the dysregulation of lncRNA NEAT1 is involved in atherosclerosis progression. Wang et al. found that NEAT1 is significantly upregulated in human myeloid leukemia mononuclear cells (THP-1) cells stimulated with oxLDL and promotes the inflammatory response by targeting miR-342-3p [59]. Trimethylamine N-oxide (TMAO) improves atherosclerosis by regulating the functions of endothelial cells. Wu et al. found that NEAT1 is upregulated in TMAO-stimulated HUVECs and plays an important role in regulating cell proliferation [60]. Zhang et al. reported that NEAT1 is upregulated in the serum of AS patients and in oxLDL-stimulated HAECs and that the suppression of NEAT1 inhibits human aortic endothelial cells (HAECs) proliferation and promotes apoptosis by targeting miR‑638 to regulate the AKT/mTOR pathway [61]. Recently, NEAT1 expression was reported to be increased in AS clinical samples and TMAO-treated HUVECs, and NEAT1 suppression suppressed proliferation and induced apoptosis in HUVECs [62]. Mechanistically, NEAT1 modulates STAT3 expression by binding to miR-370-3p and attenuates HUVEC functions during AS progression.

The lncRNA RMST has been revealed to play a crucial role in cerebral ischemic disease, and the suppression of RMST expression protects against ischemic brain injury [63, 64]. Recently, RMST was shown to play a critical role in regulating EC function in AS [65]. RMST is upregulated in the serum of AS patients, and oxLDL-induced HUVECs and RMST depression are involved in cell viability and the inflammatory response by targeting miR-224-3p. Furthermore, the receiver operating characteristic (ROC) curve indicated that RMST has clinical diagnostic value for AS and that RMST could serve as a potential diagnostic marker for AS [65].

LncRNA RNCR3, known as LINC00599, was first shown to be dysregulated during mouse retinal development and was subsequently reported to play an important role in regulating the differentiation of neurons and oligodendrocytes [66, 67]. Importantly, the expression of RNCR3 is upregulated in human aortic atherosclerotic lesions and oxLDL-induced ECs and VSMCs [68]. Furthermore, RNCR3 knockdown inhibits proliferation and migration and promotes apoptosis of ECs and VSMCs by inhibiting miR-185-5p. Recently, Hong et al. reported that the expression of RNCR3 is increased in the serum of atherosclerosis patients and that RNCR3 overexpression improves proliferation and the production of inflammatory factors, such as IL-6, IL-1β, and TNF-α, in ECs [69]. Mechanistic studies revealed that RNCR3 binds to miR-185-5p, regulates cyclin D2 expression, and promotes cell growth and cytokine secretion, which suggests that RNCR3 may be a potential target for atherosclerosis treatment.

LncRNA SNHG12, a tumor activator in cancers, was upregulated in brain microvascular ECs in a model of ischemic stroke, and it could serve as a biomarker of pulmonary arterial hypertension [70–72]. Importantly, SNHG12 was shown to play a regulatory role in the apoptosis and proliferation of oxLDL-stimulated VSMCs [73]. Recently, the modulatory role of SNHG12 in ECs of AS was investigated. Mao et al. found that SNHG12 is upregulated in AS patients and that oxLDL-stimulated HUVECs and SNHG12 can promote proinflammatory factor secretion and augment atherosclerotic lesions in vivo via the miR-218-5p/IGF2 axis [74].

LncRNA NORAD, a newly discovered noncoding RNA located at Chr20q11.23, can be activated via DNA damage and is related to maintaining genomic stability [75]. NORAD transcripts have been found to function both in the cytoplasm and nucleus [76]. In the cytoplasm, NORAD targets PUMILIO and modulates cell proliferation and division by regulating mRNA stability [77]. In addition, NORAD is involved in maintaining mitochondrial homeostasis by regulating PUMILIO. Recently, NORAD was found to play a critical role in atherosclerosis progression by regulating endothelial cells [75]. NORAD downregulation leads to cell cycle arrest in G0/G1 phase, promoting senescence and apoptosis in oxLDL-stimulated HUVECs. Mechanistically, NORAD causes endothelial cell senescence and apoptosis in atherosclerosis, mainly by manipulating the NF-κB and p53–p21 pathways and IL-8 expression, in which NORAD-mediated regulation of IL-8 expression may directly target splicing factor proline and glutamine rich (SFPQ).

LncRNA SNHG12, which is involved in promoting the proinflammatory response and augmenting atherosclerotic lesions, also regulates vascular endothelium senescence during AS [78]. Hemmig et al. reported that SNHG12 is highly expressed in vascular ECs and downregulated during lesion progression and that SNHG12 knockdown improves atherosclerotic lesion formation by exacerbating DNA damage and senescence in vascular ECs.

Epithelial-mesenchymal transition (EMT) is a key contributor to atherosclerosis progression, and lncRNAs are widely related to atherosclerosis development. The lncRNA ZFAS1, located on the antisense strand of the promoter region of ZNFX1, was first discovered in breast cancer and then shown to play an important role in oncogenic properties in other cancers [79, 80]. Recently, ZFAS1 was demonstrated to be related to the process of atherosclerosis and involved in regulating macrophage functions during the progression of atherosclerosis [81]. In addition, ZNFX1 was investigated and reported to be involved in EMT during atherosclerosis [82]. The expression of ZFAS1 is significantly increased in atherosclerotic mice, and oxLDL-induced HUVECs and ZFAS1 overexpression in HUVECs promote EMT-related gene expression, including α-SMA and vimentin. Mechanistic studies have shown that ZFAS1 promotes EMT mainly by targeting the miR-150-5p/Notch3 signaling pathway.

The lncRNA ANRIL, a 3.8-kb transcript from the INK4B‐ARF‐INK4A gene cluster located in the chromosome 9p21 region, is involved in a variety of diseases, such as diabetes and coronary heart disease [83, 84]. ANRIL has been documented to play a key role in the development of AS. Liu reported that the level of ANRIL in blood plasma is significantly upregulated in acute coronary syndrome patients and that the expression level of ANRIL is positively related to the inflammatory cytokine production level [85]. In addition, ANRIL knockdown significantly promotes tubule formation and cell proliferation and suppresses inflammatory secretion by HUVECs. Furthermore, ANRIL mediates HUVEC dysfunction mainly by regulating the TGF-βR1/Smad pathway and suppressing let-7b expression. In addition, ANRIL also affects the progression of intracranial aneurysm (IA) by manipulating VSMCs. Hu et al. found that ANRIL levels were decreased in the plasma and arterial wall of IA patients and that overexpression of ANRIL promoted proliferation and blocked apoptosis of VSMCs [86]. The above results reveal that ANRIL may play a variety of regulatory roles in cardiovascular disease.

Bai et al. analyzed a Gene Expression Omnibus (GEO) dataset and found that lncRNA AK136714 was upregulated in the plaque and plasma of atherosclerosis patients [87]. Furthermore, they found that AK136714 knockdown maintained the endothelial barrier, suppressed EC inflammation and alleviated atherosclerosis formation in ApoE^−/−^ mice. Mechanistic studies have verified that AK136714 directly targets HuR (ELAVL1) to promote the mRNA stability of TNF-α and IL-1β and increases the transcription of Bim, indicating that AK136714 could function in atherosclerosis and provide potential novel drug targets for atherosclerosis intervention.

### Role of circRNAs in endothelial cells

CircRNAs have been revealed to play a key regulatory role in the occurrence and development of AS, and dysregulated circRNAs have been found in ECs during the AS process. CircRSF1, known as circ_0000345, derived from remodeling and spacing factor 1 (RSF1), was found to be significantly upregulated in HUVECs induced by oxLDL through a circRNA microarray assay, and knockdown of circ_0003575 improved the proliferation and angiogenesis of HUVECs [88]. Furthermore, Wei et al. found that circRSF1 expression was downregulated in the serum of AS patients and oxLDL-induced HAECs and that upregulating circRSF1 promoted cell viability, migration, and tube formation of oxLDL-treated HAECs and inhibited cell apoptosis [89]. Mechanistically, circRSF1 competitively targets miR-758 and positively regulates the expression of CCND2, indicating that circRSF1 could be a potential future target for atherosclerosis treatment. Recently, Zhang et al. also found that circRSF1 was downregulated in oxLDL-treated HUVECs and that overexpression of circRSF1 could promote cell proliferation and inhibit apoptosis and inflammation in oxLDL-stimulated HUVECs [90]. Further study confirmed that circRSF1 could bind directly to miR-135b-5p and regulate histone deacetylase 1 (HDAC1) expression in AS progression. These investigations of circRSF1 in AS indicated the potential therapeutic role of circRSF1 in the diagnosis and therapy of AS.

CircNOL12, known as circ_0004543, is located on chr22 and is derived from the nucleolar protein 12 (NOL12) gene. Recently, the regulatory role of circNOL12 in the occurrence and development of AS was investigated. CircNOL12 expression is increased in oxLDL-stimulated HUVECs, and downregulation of circNOL12 significantly improves the proliferation, migration, and invasion of HUVECs and inhibited their apoptosis induced with oxLDL. Furthermore, circNOL12 knockdown can activate the PI3K/AKT/NOS3 signaling axis in oxLDL-treated HUVECs, which provides a potential target for treating EC injury in AS [91]. Kaempferol (Kae), a natural alternative flavonoid, has been used in several diseases, including cancer and cardiovascular diseases, due to its antioxidant and anti-inflammatory activities [92, 93]. Importantly, Kae has been documented to suppress apoptosis of HAECs stimulated by oxLDL by suppressing the NF-κB signaling pathway [94]. Li et al. reported that Kae could attenuate oxLDL-stimulated circNOL12 upregulation and alleviate oxLDL-induced inflammation and apoptosis in HUVECs [95]. They also found that circNOL12 could bind directly to miR-6873-3p in oxLDL-treated HUVECs.

Phosphofurin acidic cluster sorting protein 2 (PACS2) expression is increased in oxLDL-treated HUVECs, and PACS2 upregulation aggravates oxLDL-induced EC apoptosis [96]. Recently, it was found that circ_0033596 expression, originating from the PACS2 transcript, is enhanced in oxLDL-treated HUVECs, and circ_0033596 overexpression is able to suppress HUVEC viability and cell cycle progression and improve apoptosis [97]. Further studies have demonstrated that circ_0033596 targets and negatively regulates miR-217-5p expression, promotes oxLDL-stimulated HUVEC apoptosis and participates in the pathogenesis of AS.

The newly discovered circCHFR, known as circ_0029589, is located on chromosome 12 and is significantly upregulated in oxLDL-treated VSMCs according to microarray assay data [98]. Furthermore, the researchers found that circCHFR plays a critical regulatory role in the proliferation and migration of VSMCs in AS by manipulating the FOXO1/Cyclin D1 signaling pathway by targeting miR-370. Li et al. reported that circCHFR is also involved in regulating HUVEC function during AS [99]. It was found that circCHFR expression is increased in AS patients and oxLDL-induced HUVECs, and circCHFR knockdown improves viability and attenuates apoptosis of HUVECs. Mechanistic studies have revealed that circCHFR, as a molecular sponge, binds to miR-15b-5p and regulates growth arrest and DNA damage inducible gamma (GADD45G) expression in HUVECs, leading to AS progression.

Wu et al. analyzed the profile of circRNAs in young and senescent ECs by applying RNA sequencing, and they found that circGNAQ (circ_0006459), which is enriched in vascular ECs, was significantly reduced in senescent ECs [100]. Further mechanistic studies indicated that circGNAQ may serve as a sponge to manipulate Polo-like kinase 2 (PLK2) expression by targeting miR-146a-5p, thereby attenuating EC senescence. Additionally, overexpression of circGNAQ in ECs weakens vascular EC senescence and reduces atherosclerosis progression, suggesting that the manipulation of circGNAQ is a potential therapeutic target for suppressing AS progression in the future.

Fu et al. performed a circRNA sequencing of peripheral blood mononuclear cells from coronary heart disease (CHD) patients and controls, and found circ_0030042, derived from forkhead box O1 (FOXO1), was significantly downregulated [101]. Furthermore, circ_0030042 plays a critical role in regulating HUVEC autophagy by targeting eukaryotic translation initiation factor 4A3 (eIF4A3) and attenuating its recruitment to the mRNA of beclin1 and forkhead box O1 (FOXO1). Importantly, circ_0030042 also promotes plaque stability and attenuates eIF4A3-stimulated plaque instability (Table 1).

**Table 1:** Summary of AS-related noncoding RNAs in endothelial

Noncoding RNAs	Effect on AS	Targets/pathway	Model	Regulation	Phenotype	Intervention	References
miR-146a	Anti-AS	Src	HUVECs	Down	Senescence↓	No	[35]
miR-216a	Pro-AS	Smad3	HUVECs/HAECs	Up	Senescent↑	Rb2	[38]
miRNA-146a-5p	Anti-AS	TRAF6/IRAK1	HUVECs	Down	Inflammation↓	No	[39]
miR-520b	Anti-AS	NF-κB/p65/ICAM1/VCAM1	HUVECs/HAECs	Down	Inflammation↓	No	[40]
miR-19b	Anti-AS	PPARγ	HUVECs	Down	Inflammation↑	No	[42]
miR-1	Pro-AS	KLF4	HUVECs	Up	Inflammation↑	No	[43]
miR-15a-5p	Anti-AS	CX3CL1	HUVECs/ApoE^−/−^ mice	Down	Proliferation↑	No	[45]
miR-155	Pro-AS	SERPIND1	HUVECs	Up	Permeability↑, proliferation↓	No	[47]
miR-512-3p	Pro-AS	XBP-1	HUVECs/ApoE^−/−^ mice	Up	Viability↑, apoptosis↓, migration↑	No	[49]
miR33a-5p	Anti-AS	MiR33a-5p/Ets-1/DKK1	HUVECs/ApoE^−/−^ mice	Down	Angiogenesis↓	No	[52]
miR-181b-5p	Anti-AS	STAT-3	HUVECs	Down	pyroptosis↓	No	[53]
miR-181b	Anti-AS	PPARδ	ApoE^−/−^ mice	Down	NOX2↓, ROS↓	Butyrate	[56]
lncRNA PVT1	Pro-AS	miR-30 c-5p	AS patients/HUVECs	Up	Proliferation↑, apoptosis↓, inflammation↑	No	[57]
lncRNA NEAT1	Pro-AS	miR-370-3p/STAT3/FMO3	AS patients/HUVECs	Up	Proliferation↑, apoptosis↓	No	[62]
lncRNA RMST	Pro-AS	miR-224-3p	AS patients /HUVECs	Up	Viability↓, inflammation↑	No	[65]
LncRNA RNCR3	Pro-AS	miR-185-5p/cyclin D2	AS serum/HUVECs	Up	Proliferation↑, inflammation↑	No	[69]
LncRNA SNHG12	Pro-AS	miR-218-5p/IGF2	HUVECs/ApoE^−/−^ mice	Up	Inflammation↑, senescence↓	No	[74] [78]
lncRNA NORAD	Anti-AS	NF-κB/p53-p21	HUVECs/ApoE^−/−^ mice	Down	Apoptosis↓, senescence↓, inflammation↓	No	[75]
lncRNA ZFAS1	Pro-AS	miR-150-5p/Notch3	HUVECs/ApoE^−/−^ mice	Up	EndMT↑	No	[82]
lncRNA ANRIL	Pro-AS	let-7b/TGF-βR1	AS patients/HUVECs	Up	Inflammation↑, apoptosis↑, proliferation↓, tubule formation↓	No	[85]
circRSF1	Anti-AS	miR-135b-5p/HDAC1	HUVECs	Down	Proliferation↑, apoptosis↓, inflammation↓	No	[90]
circNOL12	Pro-AS	miR-6873-3p/FRS2	HUVECs	Up	Inflammation↑, oxidative stress↑, and apoptosis↑	Kaempferol	[91, 95]
circ_0033596	Pro-AS	miR-217-5p/CLIC4	HUVECs	Up	Viability↓, cell cycle↓, apoptosis↑	No	[97]
circCHFR	Pro-AS	miR-15b-5p/GADD45G	AS patients/HUVECs	Up	Apoptosis↑, viability↓	No	[99]
circGNAQ	Anti-AS	miR-146a-5p	HUVECs/HCAECs	Down	Senescence↓, proliferation↑, angiogenesis↑	No	[100]
circ_0030042	Anti-AS	eIF4A3	HUVECs/ApoE^−/−^ mice	Down	Autophagy↓, plaque stability↑	No	[101]

↑: Promote; ↓: Inhibit; Up: Upreulation; Down: Downregulation

## Noncoding RNA regulation of smooth muscle cells

### Role of miRNAs in smooth muscle cells

VSMCs also play key and complex roles in the progression of atherogenesis. Under normal conditions, VSMCs are mainly quiescent and express signature markers (such as smooth muscle cell myosin heavy chain, SM22α/tagln, smooth muscle cell actin, smoothelin). However, in response to pathological signals that occur during atherogenesis, VSMCs reduce the expression of their signature markers and acquire a proatherogenic phenotype with an increased capacity for proliferation, migration and secretion. The phenotypic switching of VSMCs has been considered a key process in atherosclerosis, and intervention investigations to prevent phenotypic switching are of great significance for AS treatment. Recently, a series of studies have indicated that noncoding RNAs are critical regulators of VSMC function during AS. Gorur et al. analyzed the expression profiles of miRNAs in the plasma of AS patients and healthy controls and found that the expression of miR-222-5p was significantly upregulated in AS patients [102]. Then, Liu et al. characterized the potential regulatory role of miR-222-5p in AS progression [103]. They found that the expression of miR-222-5p in oxLDL-stimulated VSMCs and serum from AS model mice was significantly increased and that knockdown of miR-222-5p suppressed the proliferation and migration phenotype of oxLDL-stimulated VSMCs; additionally, the regulatory effect on VSMC behaviors could be reversed by knockdown of RB transcriptional corepressor 1 (RB1). Mechanistic studies have revealed that miR222-5p can target RB1 by binding to the 3ʹ-untranslated region. In addition to the regulatory role of miR-222-5p in VSMCs, the dysregulated expression of miR-222-5p in serum is also involved in lipid deposition, which suggests that miR-222-5p may serve as a novel therapeutic target for AS in the future.

It has been documented that miR-378c is dysregulated in several cancers and plays a key role in regulating tumor development [104]. Recently, miR-378c has been reported to be involved in the progression of AS. The expression of miR-378c is significantly downregulated in AS plaques and serum of acute coronary syndrome (ACS) patients compared with controls, and miR-378c knockdown facilitates VSMC phenotypic switching during atherosclerosis [105]. Samd1 can bind (Low Density Lipoprotein) LDL on the cell surface, promote the oxidation of LDL and play a critical role in foam cell formation. Furthermore, Samd1 was found to be inhibited by miR-378c, and its protein level is increased in blood from ACS patients, indicating that the miR-378c-Samd1 axis participates in both VSMC phenotypic switching and LDL oxidation during AS and may be a potential target for AS treatment.

In previous work, miR-205-5p was shown to negatively regulate the expression of low-density lipoprotein receptor-related protein 1 (LRP1), leading to cholesterol accumulation within large arterial walls because of the attenuation of the LRP1/ABCA1 pathway [106]. Meng et al. utilized miR-205-5p knock-in (KI) mice crossed with apolipoprotein E knockout (ApoE^−/−^) mice to investigate the role of miR-205-5p in atherosclerotic plaque formation [107]. It was found that miR-205-5p KI ApoE^−/−^mice develop larger, more unstable plaques than ApoE^−/−^ mice, with lower serum levels of high-density lipoprotein cholesterol. These results suggest that miR-205-5p is related to unstable plaques and has a negative influence on lipid metabolism in AS. Huang et al. also found that miR-205–5p is downregulated in an AS model and may serve as a potential biomarker of cardiovascular disease [108]. Recently, miR-205–5p was shown to inhibit the viability of human aortic vascular smooth muscle cells (HAVSMCs) stimulated by oxLDL, suppress the cell cycle by inhibiting the expression of cyclin D1, and improve cell apoptosis by enhancing the expression of Bax/Bcl-2 and caspase3 [109]. Mechanistically, miR-205-5p inhibits the proliferation and migration of oxLDL-treated HAVSMCs by attenuating the phosphorylation of (erb-b2 receptor tyrosine kinase 4) ERBB4 and AKT, suggesting that miR-205-5p may serve as a novel target for AS intervention.miR-126-3p, located in the intron of EGF-like domain multiple 7 (EGFL7), is mainly derived from ECs [110]. It has been demonstrated that miR-126-3p plays an important antiatherogenic role by modulating a variety of pathways. For example, miR-126-3p derived from EC apoptosis bodies suppresses the production of regulator of G-protein signaling 16 (RGS16) to activate CXCL12 and its receptor CXCR4, weakening atherosclerosis [111]. miR-126-3p also prevents the adhesion of leukocytes to ECs by downregulating the expression of vascular cell adhesion molecule-1 (VCAM-1) [112]. The stimulation of VSMCs with EC-derived microparticles (EMPs) rich in miR-126-3p inhibits SMC proliferation by regulating LDL receptor-related protein 6 (LRP6) and β-catenin expression [113]. Vascular calcification has an effect on aortic rigidity and can enhance the possibility of atherosclerotic lesion rupture. Recently, Zeng et al. declared that miR-126-3p is involved in the reduction of vascular calcification of SMCs [114]. Furthermore, they revealed that the upregulation of miR-126-3p in VSMCs can inhibit ERK1/2 and reduce calcification in AS lesions.

VSMC senescence causes cell dysfunction and improves the occurrence of aging-related cardiovascular diseases, including AS, where suppression of VSMC senescence could be one of the interactions in AS. Recent studies have revealed that miRNAs are essential regulators of VSMC cellular senescence. Hypoxia plays an important role in promoting VSMC proliferation. The expression of miR-665 is upregulated in PASMCs under hypoxic conditions, and the upregulation of miR-665 PASMC enhances proliferation [115]. In addition, the miR-665 level is significantly increased in VSMCs with bleomycin (BLM)-stimulated senescence and olmesartan reduced BLM-induced senescence by upregulating Syndecan 1 (SDC1), which is mediated by miR-665 [116]. Recently, Chen et al. analyzed differentially expressed miRNAs in young and old VSMCs through microarray analysis and found that miR-665 was significantly upregulated in aging VSMCs, and miR-665 knockdown attenuated VSMC senescence [117]. Further investigation demonstrated that miR-665 could promote senescence in AS mainly by targeting SDC1 in VSMCs, which may elucidate a novel intervention strategy for aging-related AS.

### Role of lncRNAs in smooth muscle cells

lncRNAs are dysregulated during vascular pathology and are involved in the switch of SMCs to a dedifferentiated state in the microenvironment of AS lesions. LncRNA CARMN, which was previously found in human cardiomyocytes, is juxta-located upstream of miR-143 and miR-145, both of which are related to regulating vSMC function [118]. Dong et al. reanalyzed large-scale single-cell RNA sequencing datasets and showed that CARMN, which had been documented to play a key role in cardiac differentiation, is an abundant, conserved, and SMC-specific lncRNA [119]. They found that CARMN expression is significantly downregulated in humans and murine models of vascular disease and regulates the phenotype of VSMCs in vitro. In vivo, specific deletion of CARMN in SMCs dramatically aggravates injury-stimulated neointima formation in mice. Mechanistic studies have revealed that CARMN targets the transcriptional cofactor myocardin, promoting its activity and thereby protecting the contractile phenotype of VSMCs. In addition, this was the first lncRNA discovered to target myocardin. Vacante et al. found that CARMN negatively regulates miR-143 and miR-145 expression in human coronary arterial smooth muscle cells (hCASMCs), and transcriptomic data from CARMN-knockout hCASMCs showed that CARMN is involved in the proliferation, migration, inflammation, lipid metabolism and dedifferentiation of SMCs [120]. Importantly, the expression of miR-143 and miR-145 is reduced after CARMN knockout, and the volume and size of plaques are increased in AS, revealing that lncRNA CARMN serves as a regulator to facilitate vSMC differentiation toward a proatherogenic phenotype and aggravates AS progression.

Differentiation antagonizing nonprotein-coding RNA (DANCR) has been documented to participate in the occurrence of a series of cancers, including lung and colorectal cancers [121, 122]. In addition, Zhang et al. discovered that DANCR is involved in AS progression by manipulating ATP binding cassette subfamily A member 1 (ABCA1), suggesting that DANCR plays a crucial role in AS development [123]. Recently, An et al. found that the expression of DANCR was upregulated in serum from AS patients and positively correlated with the levels of low-density lipoprotein cholesterol (LDL-C), homocysteine (Hcy), and C-reactive protein (CRP) in serum [124]. In addition, DANCR knockdown attenuates the proliferation and migration of VSMCs. It was further revealed that DANCR aggravates AS progression by binding to miR-335-5p. Similarly, Zhang et al. have reported that DANCR is dramatically upregulated in the serum of AS patients and oxLDL-stimulated VSMCs and HUVECs [125]. In addition, DANCR downregulation significantly enhances viability and suppresses apoptosis of oxLDL-treated VSMCs. Moreover, DANCR downregulation inhibits the expression of inflammatory cytokines, such as IL-6, IL-1β, and TNF-α, in oxLDL-treated VSMCs and HUVECs. Mechanistically, DANCR can regulate the expression of cytochrome c oxidase assembly factor COX20 (COX20) by binding to miR-214-5p, and the downregulation of miR-214-5p can weaken the protective effects of si-DANCR on oxLDL-induced EC injury. These results demonstrate that DANCR may be a potential target for AS intervention.

LncRNA XIST, derived from the inactivated X chromosome, is related to X chromosome inactivation in female mammals, supplying compensation for the imbalance of the X-associated gene dosage between the sexes [124]. It has been documented that XIST plays an essential role in vascular diseases, including the aggravation of myocardial infarction, via miR-101a-3p-targeted fos proto-oncogene (FOS) manipulation [126]. Oxygen–glucose deprivation (OGD) upregulates the expression of XIST, and overexpression of XIST exacerbates OGD-stimulated neuronal injury by regulating the miR-455-3p/TIPARP pathway [127]. In addition, downregulation of lncRNA XIST suppresses oxLDL-induced EC inflammation and apoptosis by regulating the expression of miR-221-3p [128]. Recently, the regulatory role of XIST, located on human chromosome Xql3.2, was investigated in oxLDL-treated VSMCs. Zhang et al. found that XIST was upregulated in oxLDL-treated VSMCs and that knockdown of XIST limited the proliferation and migration of oxLDL-stimulated VSMCs [129]. XIST can directly target miR-539-5p and suppress its expression. In addition, inhibition of miR-539-5p expression can partially reverse the effect of XIST downregulation on the migration and proliferation of oxLDL-stimulated VSMCs. A mechanistic investigation revealed that the expression of secreted phosphoprotein 1, a target of miR-539-5p, is regulated by XIST to promote the proliferation and migration of VSMCs during AS progression.

The lncRNA NRON, composed of three exons with a length of 2.7 kb, plays an important role in the assembly of a large RNA/protein complex [130]. NRON was previously thought to be related to the immune system and involved in the differentiation of T cells [130]. In addition, Nron has been reported to manipulate the pathophysiology and participate in processes involved in a series of diseases [131, 132]. Recently, Du et al. studied the role of NRON in the progression of AS and clarified the regulatory mechanism of Nron in SMC function and intraplaque angiogenesis [133]. They found that the expression of NRON is depressed in atherosclerotic lesions from humans and mice and that overexpression of NRON in VSMCs causes more fragile plaques with an increase in the degradation of elastic fibers. Conversely, NRON knockdown attenuates the progression of AS and enhances the stability of AS plaques. Mechanistic studies have revealed that NRON specifically targets and negatively manipulates nuclear factor of activated T cells 3 (NFATC3), thus suppressing the proliferation and exacerbating the apoptosis of VSMCs during the process of AS, which suggests that targeting and inhibiting NRON expression may have future potential for AS intervention. In addition, lncRNA-p21 has also been reported to play a protective role against AS development by inhibiting the proliferation and promoting the apoptosis of VSMCs by targeting sirtuin 7 (SIRT7) [134].

### Role of circRNAs in smooth muscle cells

CircRNAs generally have a long half-life by resisting exonuclease degradation. The regulatory role of circRNAs has been investigated in cancer and cardiovascular and neurologic diseases [135]. CircRNAs have been reported to be involved in vascular diseases by regulating VSMCs. CircMTO1 is conserved in mice and humans, and it has been found to be dysregulated in a series of cancers [136]. Recently, it was reported that CircMTO1 plays an essential role in regulating the function of VSMCs during AS progression [137]. Ji et al. found that the expression of circMTO1 is reduced in serum samples from AS patients and oxLDL-treated VSMCs, suggesting that circMTO1 is related to the development of AS. CircMTO1 upregulation suppresses proliferation and migration and enhances apoptosis in oxLDL-treated VSMCs. CircMTO1 was further found to regulate the expression of RAS p21 protein activator 1 (RASA1) by inhibiting miR-182-5p. Furthermore, re-expression of miR-182-5p and knockdown of RASA1 was found to reverse the effects of circMTO1 on the migration, proliferation, and apoptosis of oxLDL-treated VSMCs, indicating that circMTO1 could serve as a potential target in AS intervention.

RNA-Seq was applied to identify vascular remodeling–related circRNAs in AS tissues obtained from ApoE^–/–^ model mice. circEsyt2, the top 5 dysregulated circRNAs involved in junction counts, was significantly upregulated in plaques from the AS model and in VSMCs from damaged carotid arteries [138]. circEsyt2 knockdown decreased the proliferation of VSMCs, as measured by Ki67 staining. In addition, after circEsyt2 knockdown, expression of the phenotypic switch markers SMA and Myosin heavy chain 11 (Myh11) was elevated in the damaged carotid arteries. Furthermore, circEsyt2 knockdown promoted VSMC apoptosis in vivo. Mechanistic studies have shown that circEsyt2 can interact directly with Poly(rC) binding protein 1 (PCBP1) and regulate the intracellular location of PCBP. Furthermore, silencing of circEsyt2 significantly increases p53 splicing via PCBP1, resulting in altered expression of p53 target genes (cyclin D1, PUMA, and NOXA) and decreased VSMC proliferation. These results suggest that circEsyt2 could serve as a key target for combating AS caused by vascular remodeling.

It has been reported that circHIPK3 is involved in myocardial repair and regeneration by targeting miR-133a and enhancing the expression of connective tissue growth factor (CTGF) [139]. Recently, circHIPK3 was identified to play a crucial role in AS progression. Wei et al. reported that circHIPK3 is repressed in high fat-fed mice and oxLDL-stimulated HUVECs [140]. Furthermore, they revealed that circHIPK3 could suppress lipid content in ox-LD-stimulated HUVECs by promoting autophagy by regulating the miR-190b/ATG7 axis, which suggested that circHIPK3a is involved in AS development by manipulating VECs. In addition, circHIPK3 was also found to be upregulated in human vascular smooth muscle (HVSMCs) during AS progression [141]. The CCK-8 experiment revealed that knocking down circHIPK3 decreases the growth of VSMCs. In VSMCs, knocking down circHIPK3 causes cell cycle arrest and apoptosis. These data suggest that circHIPK3 control may be an essential regulatory factor for VSMC proliferation in AS.

Studies have investigated the relationship between dysregulated circRNAs and vascular calcification (VC). Ryu et al. performed RNA sequencing of inorganic phosphate-induced VSMCs and profiled the dysregulated circRNAs during VC progression [142]. A series of circRNAs were found and verified to be involved in the development of VC. For example, circSmoc1–2 were significantly downregulated post VC induction. Recently, the authors further confirmed that the expression of circSmoc1–2, located mainly in the cytoplasm, is decreased after VC induction. circSmoc1–2 overexpression substantially attenuates calcium deposition, implying that circSmoc1–2 modulation has an impact on VC. Furthermore, inhibition of circSmoc1–2 results in calcium deposition, but combining a miR-874-3p inhibitor with a circSmoc1–2 siRNA results in reduced calcium deposition, indicating that circSmoc1–2 acts as a sponge for miR-874-3p during VC. The above investigations revealed that circSmoc1–2 could be a new therapeutic target for the treatment of VC during AS (Table 2).

**Table 2:** Summary of atherosclerosis-related noncoding RNAs in smooth muscle cells

Noncoding RNAs	Effect	Targets/pathway	Model	Regulation	Phenotype	Intervention	References
miR-222-5p	Pro-AS	RB1	HAVSMCs/ApoE^−/−^ mice	up	Proliferation↑, migration↑	No	[103]
miR-378c	Anti-AS	Samd1	HAVSMCs	Down	Proliferation↓, migration↓	No	[105]
miR-205-5p	Anti-AS	ERBB4/AKT	HAVSMCs	Down	Proliferation↓, migration↓	No	[109]
miR-126-3p	Anti-AS	ERK1/2	HASMCs/HUVECs/ApoE^−/−^ mice	Down	Calcification↓	No	[114]
miR-665	Pro-AS	lncRNA GAS5/SDC1	HASMCs	Up	Senescence↑	No	[117]
lncRNA CARMN	Anti-AS	miR-143 and mi-R145	HASMCs/atherosclerotic plaques	Down	Proliferation↓, migration↓,	No	[120]
lncRNA DANCR	Pro-AS	miR-214-5p/COX20	HASMCs/AS patients	Up	Viability↓, apoptosis↑, Inflammation↑	No	[124, 125]
LncRNA XIST	Pro-AS	miR-539-5p/SPP1	HASMCs	Up	Proliferation↑, migration↑	No	[129]
LncRNA Nron	Pro-AS	NFATc3	HASMCs	Up	Proliferation↓, apoptosis↑	No	[133]
LncRNA-p21	Pro-AS	miR-17-5p/SIRT7	AS patients/ApoE^−/−^mice	Up	Proliferation↓, apoptosis↑	No	[134]
CircMTO1	Anti-AS	miR-182-5p/RASA1	AS patients/ HASMCs	Down	Proliferation↑, apoptosis↓	No	[137]
circEsyt2	Pro-AS	PCBP1	HASMCs/ApoE^−/−^mice	Up	Proliferation↑, migration↑, apoptosis↓	No	[138]
circHIPK3	Pro-AS	miR-637	HASMC	Up	Proliferation↑,	no	[141]
circSmoc1-2	Anti-AS	miR-874-3p/Adam19	HASMC	Down	Calcification↓	no	[143]

↑: Promote; ↓: Inhibit; Up: Upregulation; Down: Downregulation

## Conclusion future direction

Because of the ability of noncoding RNAs to modulate gene expression, they have an undeniable influence on AS initiation and development. We were able to determine the possible influence of various noncoding RNAs by connecting their target genes to the consequences of downregulating their respective protein expression. This has been found to affect the phenotypic behavior of multiple critical actors in AS, including ECs and SMCs, the dysregulation of which initiates and accelerates AS plaque formation. A number of studies have suggested that noncoding RNAs play important roles in lipid metabolism, EC dysfunction, the VSMC phenotype, reverse cholesterol transfer, and vascular inflammation in atherosclerosis. Several noncoding RNAs offer strong potential as biomarkers and intervention targets for various pathological alterations that occur during the AS process. Delivery of a cassette containing noncoding mimics or inhibitors could thus be an appealing therapeutic method for specific stages of AS and management of its consequences. However, most investigations on the link between noncoding RNAs and AS are limited to cell or blood detection. To identify the specific role of noncoding RNAs in atherosclerotic plaques, in vivo investigations are needed. In addition, it is necessary to analyze the role of the same noncoding RNA in different diseases, such as the associations between AS and cancers. In addition, the models of AS are limited for investigating the regulatory roles of noncoding RNAs, and it is necessary to explore novel models that are more fully aligned with actual AS progression, such as three-dimensional (3D) cell culture systems.

Despite these challenges, the potential of ncRNAs as promising therapeutic targets and agents for atherosclerosis treatment is increasingly recognized. Future research should focus on developing more effective and targeted ncRNA-based therapies, such as antisense oligonucleotides, small molecules, and nanoparticle delivery systems, which can specifically target dysregulated ncRNAs in atherosclerotic lesions and modulate their functions. Moreover, large-scale clinical studies are needed to validate the efficacy and safety of ncRNA-based therapies in patients with atherosclerosis and related cardiovascular disorders. Overall, a deeper understanding of the regulatory and interventional roles of ncRNAs in atherosclerosis holds great promise for the development of novel diagnostic and therapeutic strategies for this complex and multifactorial disease.

## Data Availability

Data sharing not applicable to this article as no datasets were generated or analysed during the current study.
